# Arsenic Efflux from *Microcystis aeruginosa* under Different Phosphate Regimes

**DOI:** 10.1371/journal.pone.0116099

**Published:** 2014-12-30

**Authors:** Changzhou Yan, Zhenhong Wang, Zhuanxi Luo

**Affiliations:** 1 Key Laboratory of Urban Environment and Health, Institute of Urban Environment, Chinese Academy of Sciences, Xiamen 361021, China; 2 College of Chemistry and Environment, Minnan Normal University, Zhangzhou 363000, China; University of Ottawa, Canada

## Abstract

Phytoplankton plays an important role in arsenic speciation, distribution, and cycling in freshwater environments. Little information, however, is available on arsenic efflux from the cyanobacteria *Microcystis aeruginosa* under different phosphate regimes. This study investigated *M*. *aeruginosa* arsenic efflux and speciation by pre-exposing it to 10 µM arsenate or arsenite for 24 h during limited (12 h) and extended (13 d) depuration periods under phosphate enriched (+P) and phosphate depleted (−P) treatments. Arsenate was the predominant species detected in algal cells throughout the depuration period while arsenite only accounted for no greater than 45% of intracellular arsenic. During the limited depuration period, arsenic efflux occurred rapidly and only arsenate was detected in solutions. During the extended depuration period, however, arsenate and dimethylarsinic acid (DMA) were found to be the two predominant arsenic species detected in solutions under −P treatments, but arsenate was the only species detected under +P treatments. Experimental results also suggest that phosphorus has a significant effect in accelerating arsenic efflux and promoting arsenite bio-oxidation in *M. aeruginosa*. Furthermore, phosphorus depletion can reduce arsenic efflux from algal cells as well as accelerate arsenic reduction and methylation. These findings can contribute to our understanding of arsenic biogeochemistry in aquatic environments and its potential environmental risks under different phosphorus levels.

## Introduction

Arsenic (As), ubiquitous throughout global environments [1], [2], is toxic to multicellular organisms being a group 1 carcinogen as specified by the International Agency for Research on Cancer (IARC). Arsenic is easily released through select human activities (such as mining, nonferrous metal smelting and refining, arsenical herbicide and insecticide usage, and fossil fuel combustion) and naturally through Earth’s crust [3], [4]. Elevated arsenic concentrations in freshwater not only severely impact safe drinking water provisions [5], [6] but have lately raised public concern on food safety [7], [8]. Inorganic arsenic species make up the bulk of total dissolved arsenic in freshwater [9] in nonionic trivalent (arsenite) and ionic pentavalent (arsenate) forms [10]. Arsenite has a high affinity for sulfhydryl groups of the amino acid cysteine (Cys), affecting many key metabolic processes such as fatty acid metabolism and glutathione production. Arsenate is a phosphate analogue and can substitute phosphate in adenosine triphosphate (ATP), affecting cell nucleotide synthesis and energy homeostasis [11].

Phytoplankton, being the primary producer in aquatic ecosystems, plays an important role in arsenic speciation, distribution, and cycling in freshwater environments [6]. It is well-established that certain freshwater phytoplankton species are able to oxidate/reduce, methylate, and release arsenic into external environments [12]–[14]. Recent studies by Yin et al. [13], [15] show that certain algae species (such as *Chlamydomonas reinhardtii* and *Synechocystis sp.*) rapidly uptake, transform (oxidate/reduce/methylate), and extrude arsenic to external media after only 12 h of arsenate or arsenite exposure. Moreover, changes in arsenic species caused by freshwater algae are considered to be significantly affected by phosphorus levels in aquatic environments [12], [16], [17]. Phosphate deficiency (−P), for example, markedly increases the accumulation of arsenate and methylarsenical production in algal cells as well as levels of arsenate toxicity [12], [18], [19] while phosphate abundance or excess can inhibit arsenate reduction by impeding arsenate influx into cells via phosphate transport systems or by competitively binding to the active site of ArsC (arsenate reductases) [20]. Not only is the uptake of various As species essential for the evaluation of As toxicity, depuration and efflux of aquatic organisms after exposure are vital to As migration and transformation within aquatic environments. On the one hand, it is important to understand As efflux from algae in As contaminated freshwater systems; on the other hand, little information related to arsenic efflux mechanisms in algae is presently available, particularly under different phosphate regimes.

*Microcystis aeruginosa* is one of the most common and widespread bloom-forming cyanobacteria in freshwater environments, often resulting in serious water quality issues to arise. The objective of this study was to report on a series of algal culture experiments designed to demonstrate how arsenic efflux from *M. aeruginosa* reacts in freshwater under different phosphate treatments. Changes in arsenic speciation for both the alga and culture media were also measured. Results from this study can provide a better interpretation of arsenic biogeochemistry in environments under different phosphorus regimes.

## Materials and Methods

### Organisms used and culture conditions applied

The unicellular cyanobacterium (*M. aeruginosa*, FACHB-905) selected for this study was isolated from eutrophic water in Dianchi Lake and obtained from the Freshwater Algae Culture Collection of the Institute of Hydrobiology, China. Unialgal inoculant was cultured in sterile BG-11 media under an irradiance of 115 µmol photons m^−2 ^s^−1^ with a photoperiod of 16 h light/8 h dark at 25±2°C [21]. Erlenmeyer flasks containing algae were rotated and shaken thrice daily by hand to ensure sufficient gas exchange.

### Arsenic efflux experiments

Before arsenic efflux experiments were carried out, *M. aeruginosa* (approximately 3–5×10^6^ cells mL^−1^) was precedently and individually exposed to 10 µM arsenate (Na_3_AsO_4_•12H_2_O) or arsenite (NaAsO_2_) in a 300 mL sterile nutrient solution (BG-11) for 24 h. For each treatment, 30 mL aliquots of algal inoculant were centrifuged into a pellet form before all were twice washed in sterile Milli-Q water (18.2 mΩ•cm^−2^; Millipore) and incubated in an ice-cold phosphate buffer for 10 min to remove weakly bound arsenic on cell surfaces [13]. All utensils were sterilized in autoclave, and all operations were carried out on an ultraclean workbench to ensure axenicity. After being washed in sterile Milli-Q water a second time, two pellets were prepared from which to detect intracellular arsenic speciation while the remainder was respectively resuspended at a concentration of 2–5×10^5^ cells mL^−1^ in 150 ml BG-11 media with or without 150 mM phosphate for depuration. Each depuration treatment was replicated in three flasks. The phosphate-depleted (−P, without any addition of phosphorus) BG-11 media and the common phosphate-enriched (+P) BG-11 media were manipulated to see how this variation might influence arsenic excretion. At the same time, alga not exposed to As were inoculated in +P and −P media with a similar cell density to act as corresponding controls for +P and −P environments, respectively.

In order to analyze arsenic efflux behavior, the algal depuration period was set to limited and extended periods of 12 h and 13 d, respectively. Throughout this algal depuration period, 10 mL algal suspension aliquots were periodically removed from solutions. To calculate the specific growth rate of , algal cell concentrations were determined by measuring optical density at a wavelength of 682 nm using a Thermo UV-Vis spectrophotometer (Thermo Evolution 300, Thermofisher Scientific, USA). Algae were subsequently centrifuged to create an algal pellet and then freeze-dried in a vacuum freeze dryer for arsenic speciation analysis. The supernatant was filtered through 0.45 µm cellulose acetate syringe filters and stored at -20°C for analysis of total arsenic and arsenic species present.

Arsenic partition coefficients (*K_d_*) between algae and aqueous phases were calculated using the following formula (where *C_a_* is the concentration in algae, and *C_w_* is the measured free concentration in aqueous BG-11 media):
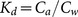
(1)


### Sample preparation and analysis

For analysis of arsenic speciation, freeze-dried alga samples were extracted through digestion in a microwave-accelerated reaction system (MARS-Xpress, CEM Microwave Technology Ltd, USA) after being soaked in 5 mL of 1% HNO_3_ (guaranteed reagent grade) overnight. The working method was as follows: 55°C for 10 min, 75°C for 10 min, and 95°C for 30 min, applying a 5 min ramp time between each stage [13], [22]. Extracts were filtered through a 0.45 µm syringe filter before arsenic speciation analysis. Arsenic species in nutrient solutions and algae extracts were determined by HPLC-ICP-MS (Agilent LC1100 series and Agilent ICP-MS 7500a; Agilent Technologies) in the manner previously described [22]. Arsenic species (arsenite, arsenate, dimethylarsinic acid (DMA), and monomethylarsonic acid (MMA)) were separated using an anion-exchange arsenic speciation column (Hamilton PRP-X100 10 µm) fitted with a matched guard column (Hamilton 11.2 mm, 12–20 µm). The mobile phase was a mixed solution of 10 mM NH_4_H_2_PO_4_ and 10 mM NH_4_NO_3_, adjusted to pH 6.2 using nitric acid or ammonia, which was isocratically pumped through the column at 1 mL min^−1^. The solution from the separation column was continuously mixed in an internal standard solution (germanium, Ge) before being introduced into the ICP-MS concentric nebulizer and a water-jacketed cyclonic spray chamber. Possible polyatomic interference of ArCl on m/z 75 was removed using the Agilent Octopole Reaction System operating in helium gas mode. Arsenic species in samples were quantified by peak areas in external calibration curves. Total arsenic concentrations in nutrient solutions were determined by ICP-MS (Agilent 7500a), operating in the helium gas mode to remove possible interference of ArCl on m/z 75.

### Quality control and data analysis

Certified reference materials (GBW 08521 Laver obtained from the National Research Center for Standard Materials in China) and blanks were included to ensure arsenic quality. Repeated analysis of certified reference materials yielded 43±1 µg As g^−1^ (mean±SD), which was similar to the certified concentration (certified value 41±3 µg As g^−1^). OriginPro 8.5.0 SR1 software (OriginLab Corporation, 1991–2010) was used to measure concentrations of each compound identified. Total arsenic quantity in algal cells was calculated as the sum of concentrations of different arsenic species in cells. All data were subjected to analysis of variance (ANOVA) using windows-based SPSS 15.0 and GraphPad Prism 6.0.

## Results

### *M. aeruginosa* sensitivity during arsenic depuration

**Figure 1:**
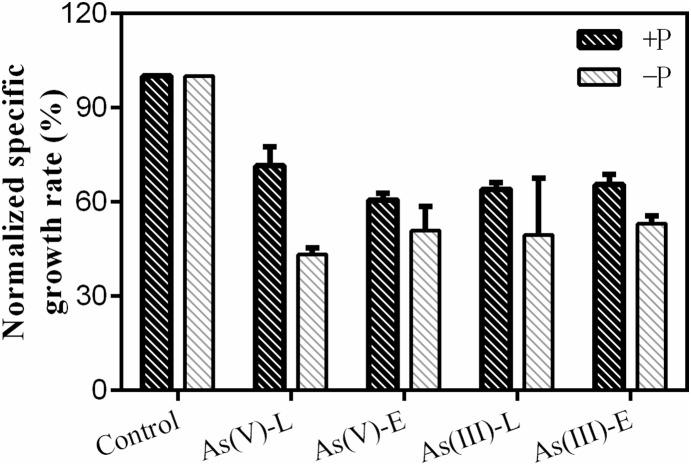
Changes in the normalized specific growth rate of *M. aeruginosa* after 24 h individual exposure to 10 µM As(V) and As(III) under different phosphorus treatments (+P or −P). Control: no arsenic added; As(V) −L and As(III) −L: limited depuration period after individual arsenate and arsenite pre-exposure; As(V) −E and As(III) −E: extended depuration period after individual arsenate and arsenite pre-exposure. Data are means ± SD (n = 3).

Changes in the specific growth rate of during the arsenic depuration period under different phosphate regimes were normalized to reflect control treatments under ±P treatments devoid of added arsenic (Fig. 1). During the 13 d recovery period (extended period), both arsenate and arsenite exhibited inhibition effects in treatments with significant decreases in relative growth rates (in +P media, arsenate: 39±2% and arsenite: 35±3%; in −P media, arsenate: 49±8% and arsenite: 47±3%). Changes in the relative growth rate also exhibited similar patterns during the 12 h recovery period (limited period). Notably, although *M. aeruginosa* was able to recuperate under different phosphate regimes after arsenate or arsenite pre-exposure, the cyanobacteria showed a greater recovery potential in +P media than in −P media, indicating that toxic mechanisms of arsenic at low concentrations were associated with ambient phosphorus concentrations.

### Arsenic efflux dynamics under different phosphorus regimes

**Figure 2:**
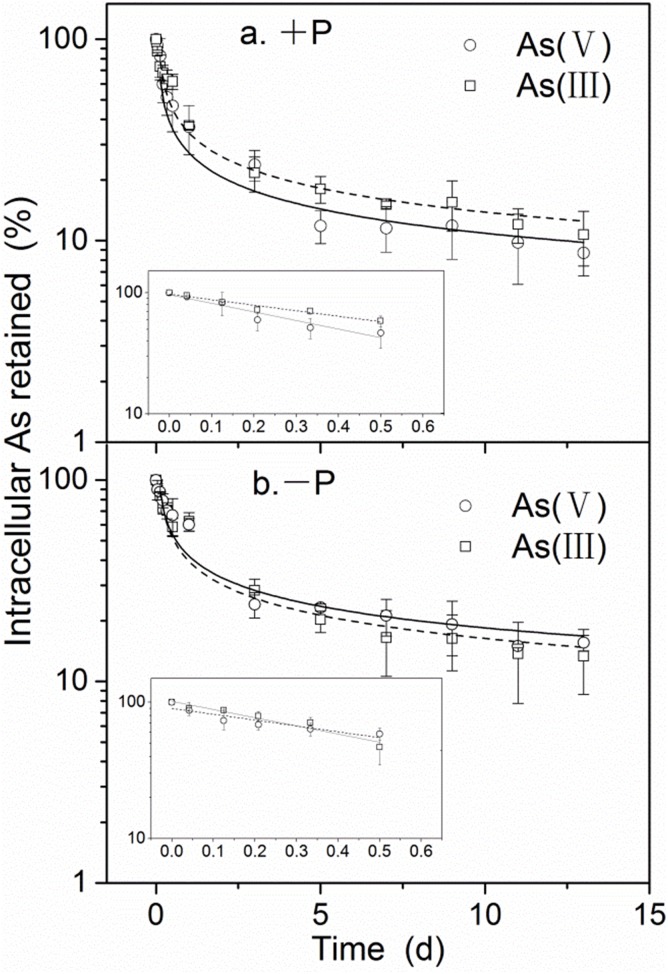
Proportional arsenic loss from *M. aeruginosa* after 24 h arsenate or arsenite exposure under the different phosphate regimes employed. Each symbol denotes arsenic concentration (from which background concentrations were subtracted) as a percentage of the intracellular concentration at 0 d (means ± SD, n = 3). Arsenic loss over a period of 13 d after a period of 24 h individual exposure to 10 µM arsenate and arsenite under +P or −P treatments is shown in (a) and (b), respectively (arsenic loss over 12 h is shown in the corresponding embedded box).

As Fig. 2 shows, the arsenic elimination period can be separated into “fast” and “slow” phases [23], [24]. For the fast phase (12 h), intracellular arsenic concentrations, expressed as the napierian logarithm (ln) of the retained proportion (%) of the initial accumulated arsenic concentration, was linear in relation to depuration time and decreased rapidly thereafter (under +P conditions, arsenate: 69% and arsenite: 53%; under −P conditions, arsenate: 60% and arsenite: 69%). However, during the slow phase, intracellular arsenic concentrations exhibited a decreasing curve pattern in relation to depuration time (1–13 d, Fig. 2). A significant effect on fast arsenic efflux was observed for both +P and −P treatments after arsenate or arsenite pre-exposure. During the slow phase, however, intracellular arsenic concentrations for both arsenate and arsenite treatments decreased by approximately 3.32% and 4.09% per day under +P and −P conditions, respectively. As anticipated, different phosphorus conditions yielded significant effects on the arsenic efflux rate.

For efflux experiments, logarithmic partitioning coefficients (log*Kd*) after arsenate pre-exposure were similar to ratios after arsenite pre-exposure. They were nevertheless lower under +P conditions than under −P conditions (Table 1).

**Table 1:** Cellular partitioning in +P or −P media.

Parameters	+P		−P	
	As(V)	As(III)	As(V)	As(III)
log*K_d_*	3.80±0.30	3.75±0.30	4.94±0.33	5.12±0.28

### Changes in cellular arsenic fractionation

**Figure 3:**
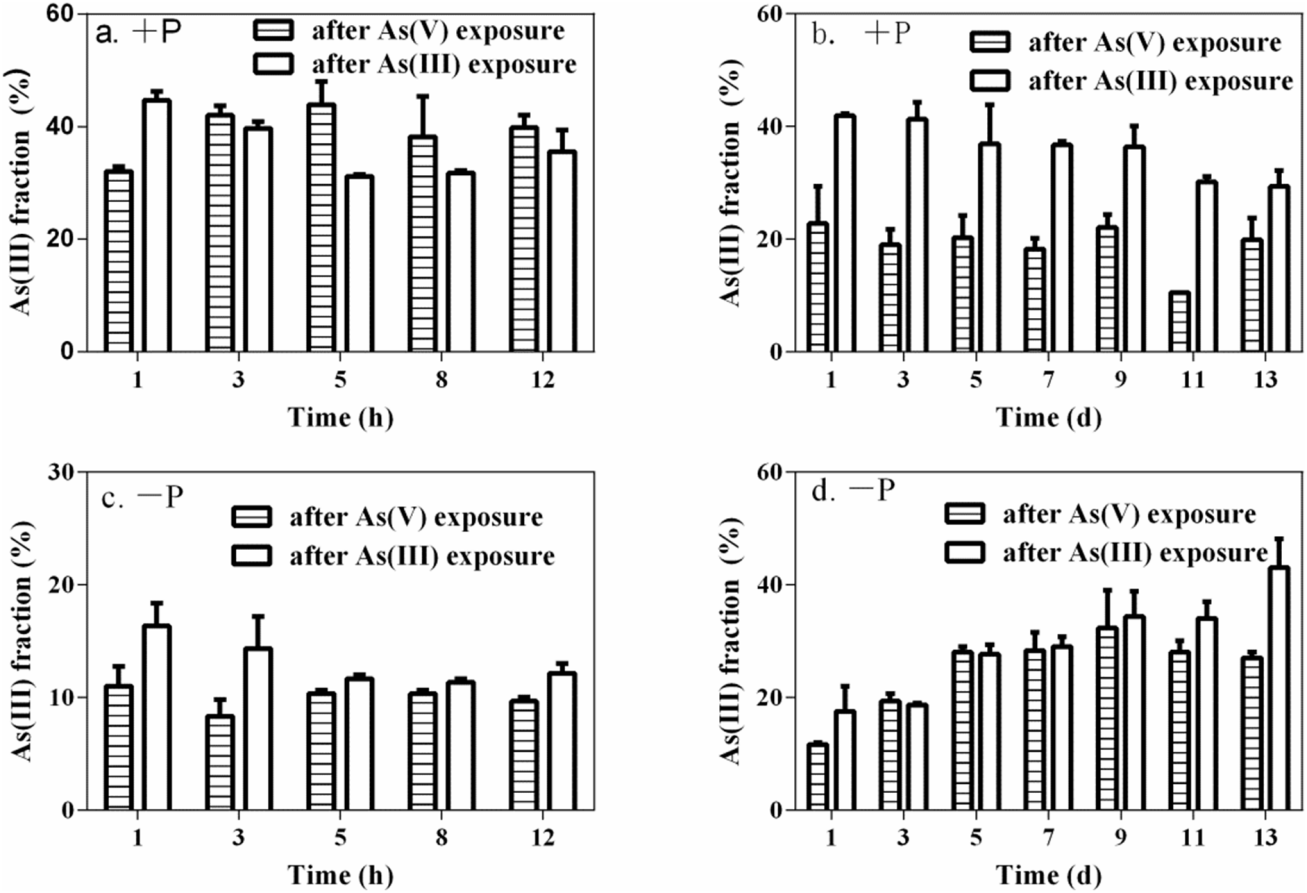
Fraction of arsenite in *M. aeruginosa* over the limited (12 h) and extended (13 d) depuration periods after 24 h of 10 µM individual arsenate and arsenite pre-exposure. The arsenite fraction under +P treatments is shown in (a) and (b). Correspondingly, the arsenite fraction under −P treatments is shown in (c) and (d). Data are means ± SD (n = 3).

*M. aeruginosa* accumulated arsenic with an average of 9.14±1.70 and 13.9±1.52 µg As g^−1^ (dw) in cells after exposure to 10 µM of arsenate and arsenite, respectively, for 24 h. Regardless of species, arsenate was the predominant arsenic species present in algae, accounting for 69±1% and 59±1% of total extractable arsenic in cells exposed to arsenate and arsenite, respectively. Throughout the entire depuration phase, moreover, arsenate was the predominant species in cells while arsenite only accounted for no greater than 45% of intracellular arsenic (Fig. 3). No methylated arsenic species were detected in algae. For the limited depuration period, arsenite percentages in algae under +P treatments were greater by a factor of two when compared to −P treatments. For the extended depuration period, arsenite percentages in algae exposed to arsenite decreased gradually over time under +P treatments, but percentages tended to exhibit a general increase over time under −P treatments (Fig. 3).

### Changes in total arsenic and its derivative species in media

**Figure 4:**
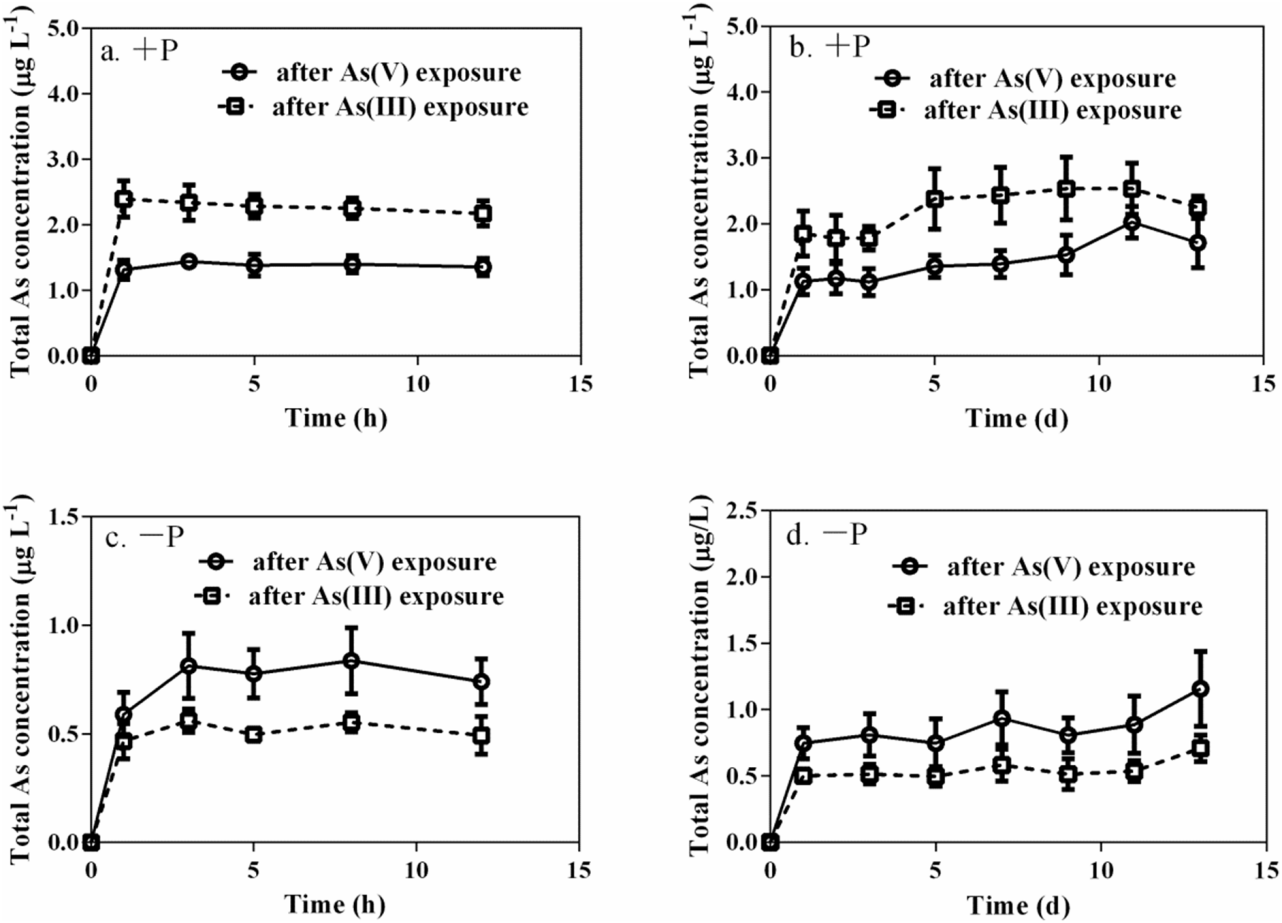
Total arsenic concentrations in solutions for the limited (12 h) and extended (13 d) depuration periods after 24 h individual 10 µM arsenate and arsenite pre-exposures. (a) and (b) represent +P treatments while (c) and (d) represent −P treatments. Each point is represented as means ± SD (n = 3).

Regardless of which arsenic species was subjected to pre-exposure, total arsenic concentrations in +P media were higher than in −P media during the arsenic efflux experiment (Fig. 4). Furthermore, for arsenite pre-exposure, total arsenic concentrations in +P media were higher by an approximate factor of 1.5 when compared to arsenate pre-exposure. For arsenite pre-exposure, in contrast, total arsenic concentrations in −P media were lower when compared to arsenate pre-exposure. It should additionally be noted that only arsenate was detected in media under +P treatments over the extended depuration period, but it was nevertheless similar to what was found in −P media over the limited depuration period.

**Figure 5:**
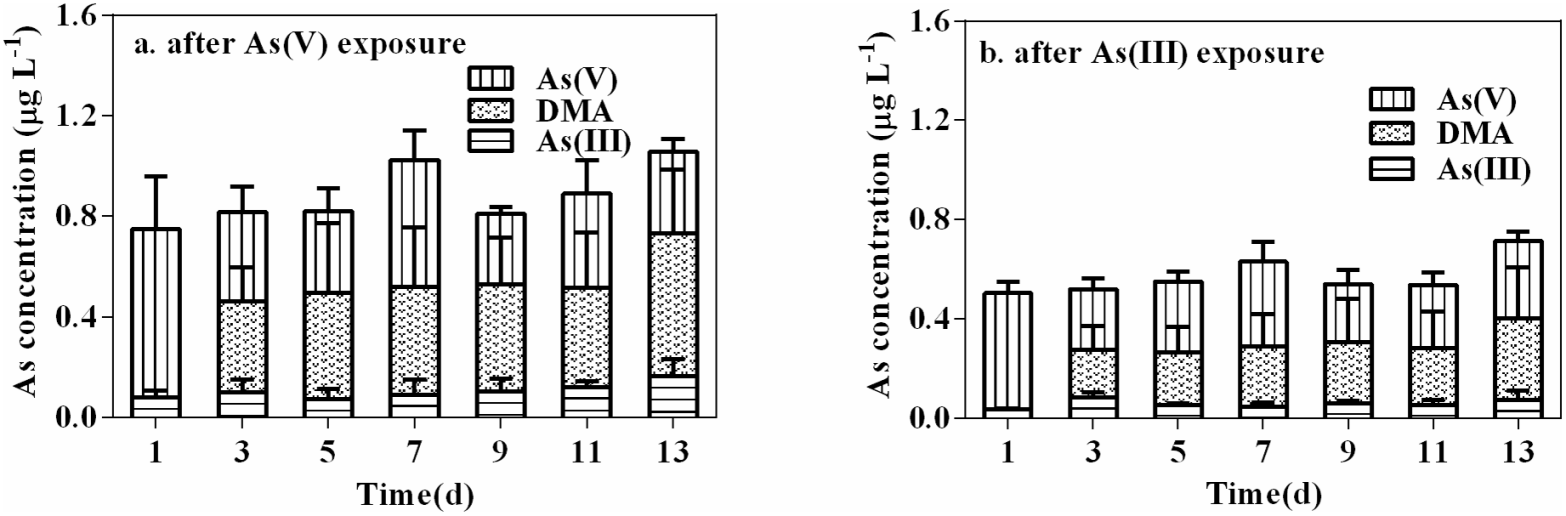
Changes in concentrations of different arsenic species in media during the 13 d depuration period under −P treatments after (a) arsenate or (b) arsenite pre-exposure. Each point is represented as means ± SD (n = 3).

As shown in Fig. 5, arsenate concentrations accounted for 89±10% and 94±10% of total arsenic for arsenate and arsenite pre-exposure experiments, respectively, after 1 d efflux in −P media and subsequently decreased to stable proportions with average ratios of 42±8% and 52±8%, respectively, for the efflux experiment days that followed. Arsenite and DMA were also detected after 12 h and 3 d, respectively, of depuration. Moreover, arsenite concentrations fluctuated and accounted for less than 14% and 16% of total arsenic in −P media for arsenate and arsenite pre-exposure experiments, respectively. DMA concentrations in −P media maintained relatively stable proportions with an average 49±5% and 40±3% for arsenate and arsenite pre-exposure experiments, respectively. These results suggest that *M. aeruginosa* possesses the capacity to methylate arsenic and, in turn, release it rapidly into ambient media.

## Discussion

This study determined that arsenic remained toxic to *M. aeruginosa*. This was supported by the fact that the growth rate of *M. aeruginosa* was significantly inhibited when algae were recovering from inorganic arsenic pre-exposure under +P and −P treatments. It is interesting to note that the specific growth rate of *M. aeruginosa* under +P treatments was clearly higher than under −P treatments, especially for the limited depuration period, suggesting that phosphorus can be used to reduce arsenic toxicity [25], [26]. Regardless of species subjected to pre-exposure (arsenate or arsenite), specific growth rates of *M. aeruginosa* during depuration experiments were similar under the same P treatments. This indicates that arsenic toxicity sensitivity to *M. aeruginosa* is related to phosphorus regimes [16].

As shown in Fig. 2, different efflux profiles were obtained during fast and slow phases. For the fast phase, intracellular arsenic concentrations were linear in relation to depuration time (12 h). However, for the slow phase (1–13 d), intracellular arsenic concentrations exhibited a decreasing curve pattern in relation to depuration time and leveled off accordingly. This type of profile, reflecting biokinetic properties, has been reported by Croteau et al. [23] and Khan et al. [24]. To date, it can be concluded that arsenic efflux rates depend not only on the species subjected to pre-exposure but also on phosphorus conditions.

The distribution of arsenic speciation in *M. aeruginosa* during the limited and extended depuration period (Fig. 3) clearly demonstrates that arsenite bio-oxidation and arsenate reduction coexist in algae. Similar findings were also observed for *Chlorella sp.*, *M. arcuatum*, and *C. reinhardtii* [15], [19]. For the limited depuration period, intracellular arsenite fractions under +P treatments were maintained at higher and steadier levels compared to −P treatments (Fig. 3), indicating that a new and rapid uptake/efflux balance was attained and that −P conditions accelerated arsenite bio-oxidation. For the extended depuration period, arsenite fractions in algae pre-exposed to arsenite decreased over depuration time under +P treatments, suggesting that bio-oxidation of arsenite to arsenate was the predominant transformation process in algal cells in freshwater enriched with arsenite and phosphate [22]. In contrast to +P treatments, the fraction of arsenite in algae increased over depuration time under −P treatments, indicating that *M. aeruginosa* possesses the capacity to biotransform arsenate into reduced arsenic species as a precursor to methylation [27], [28]. Furthermore, DMA and MMA were not detected in *M. aeruginosa* during the depuration period. Possible explanations of this are that methylated arsenic in algae was released so rapidly during the depuration period that its concentrations were lower than the detection limit. The detection of DMA and arsenite in −P media provides indirect evidence in support of this hypothesis. These results suggest that a decrease in the intracellular arsenate fraction improves the proportion of arsenite in *M. aeruginosa*, accelerating arsenic biotransformation in algae under conditions of phosphorus deficiency. Similar findings that phosphorus can regulate arsenic metabolism and speciation in rice species have also been reported [29].

In this study, only arsenate was detected in media throughout the 13 d efflux period under +P treatments, suggesting that arsenate was mainly extruded by algae under +P treatments. Inorganic arsenic species in simulated raw water were generally stable. This was because oxidation of arsenite to arsenate was inhibited in culture media [30], [31]. Previous research carried out by the authors of this study have also demonstrated that the oxidation rate of arsenite in algae-free media was slow, and only small amounts of arsenate were found in growth media containing arsenite (approximately 15%) after 15 d of incubation [22]. These results indicate that arsenate efflux is an important metabolic pathway for algal cells in solutions enriched with phosphate. It was presumed that *M. aeruginosa* can discriminate between phosphorus and arsenate and therefore rapidly excrete arsenate during the depuration period under +P treatments. Similar results have been reported [32], demonstrating that certain diatom species (*Thalassiosira spp., Chaetoceras spp.*, and *Skeletonema costatum*) appear to be able to discriminate between phosphate and arsenate, either through uptake or excretion. In addition, higher arsenic concentrations in +P media during the extended depuration period (when compared to −P media (Fig. 4)), indicate that arsenic efflux from *M. aeruginosa* is regulated by phosphorus. Lower arsenic concentrations in +P media for arsenate pre-exposure (when compared to arsenite pre-exposure) indicate that arsenic efflux from *M. aeruginosa* is not only related to phosphate concentrations in culture media but also to species type. Because arsenite pre-exposure significantly elevated intracellular arsenic concentrations (13.9±1.52 µg As g^−1^) when compared to arsenate pre-exposure (9.14±1.70 µg As g^−1^), greater quantities of arsenic (arsenate) were excreted into ambient solutions during arsenite pre-exposure [33].

For arsenate pre-exposure, however, arsenate efflux under −P treatments may be accelerated when compared to arsenite pre-exposure [34]. Similar findings have been reported for *P. vittata, C. demersum*, *Chlorella sp.*, and *M. arcuatum* [19], [35], [36]. After being transported into cells, arsenate can act as an alternative substrate of inorganic phosphate for various enzymatic reactions [37], but arseno-analogues would be unstable due to degradation by hydrolysis [38]. Additionally, Elias et al. [39] demonstrated that higher ambient ratios of P/As can improve algal capacity by discriminating arsenate over phosphate. However, lower ratios of P/As, especially under −P treatments, can delay algal capacity through arsenate and phosphate discrimination. For arsenate pre-exposure, arsenate percentages in algae under −P treatments were higher when compared to arsenite pre-exposure (Fig. 3). Therefore, arsenate was more easily extruded from algal cells via phytoplankton phosphate transport systems under −P treatments. Although DMA was lower than the detection limit and therefore went undetected in algal cells, DMA and arsenite were detected in −P media in addition to arsenate, which indicates that significant changes in arsenic species in −P media occurred over the 13 d depuration period. This suggests that DMA released from algal cells may be extremely liable. Guo et al. reported that arsenic speciation in *M. aeruginosa* culture media was more significantly affected by ambient phosphorus levels than by arsenate concentrations [16]. Significant transformations with regards to arsenic species in −P media can be explained by the fact that only small quantities of phosphorus can influence synthesis or compete with binding sites of arsenite reductase [20].

## Conclusions

In general, results from this study indicate that arsenate and arsenite are two major arsenic species that bind to *M. aeruginosa* cells during both limited and extended depuration periods under both +P or −P treatments. During the limited depuration period, arsenic efflux from *M. aeruginosa* was rapid, and only arsenate was detected in media. During the extended depuration period, both arsenate and DMA were found to be the two dominant arsenic species in media under −P treatments, but arsenate was the only species detected under +P treatments. Phosphorus has obvious effects on accelerating arsenic efflux and promoting arsenite bio-oxidation in *M. aeruginosa* after arsenate and arsenite pre-exposure. Phosphorus depletion can lessen arsenic and phosphorus discrimination for *M. aeruginosa* after arsenate pre-exposure, reduce arsenic efflux from cells, and accelerate arsenic reduction and methylation. These findings can help benefit our understanding of arsenic biogeochemistry and its potential environmental hazards under different phosphorus levels.
